# Definitive Radiotherapie versus Radiochemotherapie mit wöchentlich Docetaxel bei Patienten mit Kopf-Hals-Tumoren und Cisplatinunverträglichkeit

**DOI:** 10.1007/s00066-023-02113-6

**Published:** 2023-06-26

**Authors:** Claudia Schmalz, Jürgen Dunst

**Affiliations:** Klinik für Strahlentherapie/Radioonkologie, UKSH, Campus Kiel, Kiel, Deutschland

## Hintergrund

Bei definitiver oder adjuvanter Radiochemotherapie von fortgeschritten Plattenepithelkarzinomen der Kopf-Hals-Region ist die simultane Chemotherapie mit Cisplatin Standard auf der Basis großer Metaanalysen [4]. Schwieriger ist die Frage zu beantworten, welche medikamentöse Therapie bei Cisplatinunverträglichkeit verwendet werden sollte. Es gibt dazu keine zuverlässigen Daten. Als mögliche Alternativen werden Carboplatin, 5‑FU, Cetuximab oder Docetaxel empfohlen oder auch eine hyperfraktionierte RT [1, 5]. Allerdings muss man berücksichtigen, dass die Daten zu diesen Substanzen aus Studien mit überwiegend Cisplatin-fitten Patienten stammen. Beispielsweise wurde in der ARO-95-06-Studie die Wirksamkeit der Kombination von 5‑FU/MMC nachgewiesen [2]; bei Nierenfunktionseinschränkungen muss man aber mögliche Dosisreduktionen von 5‑FU berücksichtigen. Die jetzt publizierte indische Studie schafft Klarheit und könnte einen neuen Standard definieren [3].

## Patientengut und Methodik

Es handelte sich um eine randomisierte Phase-II/III-Studie, die am Tata Memorial Hospital in Mumbai durchgeführt wurde. Rekrutiert wurden erwachsene Patienten mit lokal fortgeschrittenen HNO-Tumoren und ECOG 0–2, bei denen einerseits die Indikation zu einer Radiochemotherapie mit Cisplatin bestand, die aber andererseits Kontraindikationen gegen Cisplatin aufwiesen. Als Cisplatinkontraindikationen wurden angesehen: Hörverlust oder Tinnitus oder neurologische Symptome Grad 2+ nach NCI-CTCAE, Kreatininclearance < 50 ml/min, unkontrollierter Hypertonus, Cisplatinüberempfindlichkeit, Malnutrition mit > 10 % Gewichtsverlust innerhalb von 6 Monaten oder BMI < 16 sowie Dauermedikation mit nephrotoxischen Substanzen. Diese Patienten erhielten entweder nur eine Radiotherapie oder eine simultane Radiochemotherapie mit Docetaxel (15 mg/m^2^ einmal wöchentlich). Die Strahlentherapie erfolgte mit werktäglich 2,0 bis zu 60 Gy adjuvant und 70 Gy bei definitiver RT. Geplant war eine Zwischenanalyse nach 356 Patienten bzw. 231 Ereignissen für den primären Endpunkt DFS mit einer erwarteten Hazard Ratio von 0,756. Danach sollte die Studie fortgeführt werden, aber wegen des bereits zu diesem Zeitpunkt nachweisbaren Effekts für das OS wurde die Studie aus ethischen Gründen bereits zu diesem Zeitpunkt beendet.

## Ergebnisse

Zwischen Juli 2017 und Mai 2021 wurden 356 Patienten rekrutiert. Die Therapieadhärenz war gut; 86 % der Patienten im Docetaxelarm erhielten wenigstens 5 wöchentliche Applikationen. Das 2‑Jahres-DFS betrug 30,3 % im RT-Arm versus 42,0 % im Docetaxel-RT-Arm (Hazard Ratio 0,67, also besser als geplant; *p* = 0,002). Das bessere DFS resultierte, wie erwartet, aus einer deutlich verbesserten lokoregionalen Tumorkontrolle (HR 0,661; *p* = 0,002). Das entsprechende mediane Gesamtüberleben (OS) betrug 15,3 Monate versus 25,5 Monate (*p* = 0,035) bzw. das 2‑Jahres-OS 41,7 % versus 50,8 % (*p* = 0,035). Bei Radiochemotherapie mit Docetaxel wurden erwartungsgemäß mehr akute Nebenwirkungen beobachtet, insbesondere eine höhere Inzidenz von Mukositis Grad 3+ (22,2 % vs. 49,7 %), Odynophagie (33,5 % vs. 52,5 %) und Dysphagie (33 % vs. 49,7 %; jeweils *p* = 0,002).

## Schlussfolgerungen der Autoren

Die zusätzliche wöchentliche Gabe von Docetaxel simultan zur Bestrahlung verbesserte das DFS und OS bei Patienten, die für eine Cisplatintherapie nicht infrage kamen.

## Kommentar

Das ist eine für die tägliche Praxis wichtige und hilfreiche Arbeit. Bisher gibt es nämlich keine belastbaren Daten zur Frage, wie man Patienten mit Kopf-Hals-Tumoren, bei denen einerseits eine Indikation für eine Radiochemotherapie mit Cisplatin und andererseits eine Kontraindikation gegen Cisplatin bestehen, am besten behandelt. Dies betrifft nicht nur die Frage, welche alternativen Medikamente als Cisplatinersatz infrage kommen, sondern auch die Bedeutung der zusätzlichen medikamentösen Therapie im Hinblick auf DFS und OS. Auf beide Fragen liefert diese indische Studie eine gute Antwort. Erstens ist das hier gewählte Regime mit Docetaxel effektiv, und zweitens entspricht die absolute Verbesserung des OS (hier immerhin 9 %-Punkte) nahezu der Wirksamkeit von Cisplatin bei günstigeren (Cisplatin-fitten) Patienten.

Einige wenige Limitationen und Kritikpunkte sollten berücksichtigt werden. Erstens wurden nur etwa 20 % der Patienten mit IMRT behandelt, wenige mit 3‑D-CRT und die mit Abstand größte Gruppe mit „konventioneller Technik“ (also 2‑D-Technik mit Gegenfeldern). Zweitens sind die jetzigen, auf einer geplanten Zwischenanalyse beruhenden Daten als präliminär anzusehen, insbesondere im Hinblick auf Spättoxizität (über die in der Publikation nicht berichtet wurde). Drittens wurden Patienten mit definitiver und adjuvanter RT hier zusammengefasst, und die Subgruppenanalyse zeigt einen besseren Effekt für die definitiv behandelte Gruppe (HR für DFS 0,58, signifikant) als für die adjuvant behandelte Gruppe (HR 0,82, n. s.). Trotz dieser Einschränkungen sind die Daten aus unserer Sicht so gut und so plausibel, dass sie als ein neuer Standard für diese klinisch immer häufiger vorkommende Konstellation angesehen werden können.

Nebenbei: Schon wieder eine „practice-changing“ Studie aus dem Tata Memorial Hospital, dem größten indischen Krebszentrum. Dass man dort offensichtlich solche Studien monozentrisch erfolgreich durchführen kann, ist beachtlich (und macht neidisch). Es unterstreicht aber auch den hohen Stellenwert, den die Radioonkologie an den indischen Krebszentren hat.

## Fazit

Eine simultane Chemotherapie mit wöchentlicher Gabe von 15 mg/m^2^ Docetaxel verbessert lokoregionale Tumorkontrolle, DFS und Gesamtüberleben bei Patienten mit Plattenepithelkarzinomen im HNO-Bereich, die für eine cisplatinhaltige Chemotherapie nicht infrage kommen.

Claudia Schmalz, Jürgen Dunst, Kiel
